# ﻿Description of a new species of *Pseudobornella* Baba, 1932 (Gastropoda, Nudibranchia, Dendronotidae) from the Yellow Sea

**DOI:** 10.3897/zookeys.1241.155540

**Published:** 2025-06-18

**Authors:** Shuqian Zhang, Juhao Wang

**Affiliations:** 1 Laboratory of Marine Organism Taxonomy and Phylogeny, Qingdao Key Laboratory of Marine Biodiversity and Conservation, Institute of Oceanology, Chinese Academy of Sciences, Qingdao 266000, China Institute of Oceanology, Chinese Academy of Sciences Qingdao China; 2 Marine Biological Museum of Chinese Academy of Sciences, Qingdao, 266071, China Marine Biological Museum of Chinese Academy of Sciences Qingdao China; 3 Shiyuan Street, Licang District, Qingdao 266000, China Unaffiliated Qingdao China

**Keywords:** Biodiversity, Chinese waters, Dendronotoidea, taxonomy

## Abstract

The genus *Pseudobornella* Baba, 1932 (Gastropoda, Nudibranchia, Dendronotidae), which was previously thought to be monotypic and restricted to the Western Pacific, is here expanded with the description of a second species, *Pseudobornellaqingdaoensis***sp. nov.**, discovered in the Yellow Sea, China. The new species is similar in general body shape to its sole congener, *P.orientalis* Baba, 1932, but these species can be distinguished based on both external morphology and internal anatomy. Phylogenetic analyses reconstructed by Bayesian inference and maximum-likelihood methods using concatenated mitochondrial (COI, 16S rRNA) and nuclear (H3) genes and species delimitation analyses support the monophyly of *Pseudobornella* as well as the separation of the two known species.

## ﻿Introduction

*Pseudobornella* was originally proposed by Baba (1932) to include *Pseudobornellaorientalis* Baba, 1932, a species of central Japan. The genus was originally placed in the family Bornellidae Bergh, 1874 (Baba 1932, 1933; Pola et al. 2009). Based on molecular evidence, Pola and Gosliner (2010) transferred *P.orientalis* to *Dendronotus* Alder & Hancock, 1845, a genus of the family Dendronotidae. This familial placement was accepted by subsequent studies, but Martynov et al. (2015) argued that *P.orientalis* could be distinguished from *Dendronotus* species by the presence of remarkably elongated papillae of the rhinophoral sheath and the absence of distinct tertiary branches of the dorsolateral appendages. Korshunova et al. (2020) presented a review of the Dendronotidae using morphological and molecular data and reinstated *Pseudobornella* as a valid genus. To date, *P.orientalis* remains the only recognized species within the genus and has a distribution spanning Japan (Baba 1932; Pola et al. 2009), China (Song et al. 2009), and the Far East of Russia (Korshunova et al. 2020).

In recent years, several nudibranch sea slugs were collected from the intertidal zone of Shandong Province, China. Through morphological study and genetic analyses, these specimens were identified as representing an undescribed species belonging to the genus *Pseudobornella*. In the present study, we describe the species and compare it with *P.orientalis*.

## ﻿Materials and methods

### ﻿Sampling and preservation

Ten specimens of the new species were collected from the intertidal zones of Rushan and Qingdao in Shandong Province, China. The live animals were photographed and preserved in 99% ethanol for molecular study and 4% formalin for morphological dissections. Type specimens were deposited at the Marine Biological Museum, Chinese Academy of Sciences (**MBMCAS**).

### ﻿Light and scanning electron microscopy

External morphology and internal anatomy were examined, dissected, and photographed using a dissecting microscope (ZEISS Discovery V20). For scanning electron microscopy (SEM) analysis, jaws and radulae were dissected from the buccal bulb. They were then cleaned with 10% NaOH, rinsed in distilled water, air-dried, coated with gold, and examined under SEM at an accelerating voltage of 5 kV.

### ﻿Molecular analyses

Six individuals of the new species and two specimens of *Pseudobornellaorientalis* were subjected to molecular analysis. Genomic DNA from each individual was extracted using the Column Genomic DNA Isolation Kit (Beijing TIANGEN, China) following to the manufacturer’s instructions. Polymerase chain reactions (PCRs) were conducted in a total volume of 25 μl, including 2 μl DNA template, 0.5 μl of each 10 mM primers, 0.5 μl of 10 mM dNTPs, 2.5 μl of 10× buffer, and 0.5 U Taq DNA polymerase. Thermal cycling was performed under the following conditions: 94 °C for 2 min (initial denaturation); followed by 30 cycles of 94 °C for 30 s (denaturation), 42 °C for 45 s (annealing), and 72 °C for 60 s (extension); and a final extension at 72 °C for 5 min. The cytochrome c oxidase subunit I gene (COI) was amplified by polymerase chain reaction (PCR) using the primers LCO1490 (forward: 5′-GGTCAACAAATCATAAAGA TATTGG-3′) and HCO2198 (reverse: 5′-TTAACTTCAGGGTGACCAAAAAATCA-3′) (Folmer et al. 1994), the 16S ribosomal RNA (16S rRNA) was amplified by the primers 16Sar (forward: 5’-CGCCTGTTTATCAAA AACAT-3’) and 16Sbr (reverse: 5’-CTCCGGTTTGAACTCAGATCA-3’) (Palumbi 1996), and the histone 3 gene (H3) was amplified by the primers HexAF (forward: 5’-ATGGCTCGTACCAAGCAGACGGC-3’) and HexAR (reverse: 5’-ATATCCTTGGGCATGATGGTGAC-3’) (Colgan et al. 1998). PCR products were verified on a GelGreen-stained 1.5% agarose gel and sequenced using the BigDye Terminator Cycle Sequencing Kit (v. 3.1 Applied Biosystems, USA) and an AB PRISM 3730 (Applied Biosystems, USA) automatic sequencer.

### ﻿Phylogenetic analyses

Three partial gene sequences (COI, 16S, and H3) were obtained from each specimen and deposited in the GenBank. Additionally, sequences of other dendronotid species were retrieved from GenBank and used for phylogenetic analyses (Table 1). Two tritoniid species, *Marioniablainvillea* (Risso, 1818) and *Marioniopsisarborescens* (Bergh, 1890), were used to root the phylogenetic tree.

**Table 1.** List of representatives of the family Dendronotidae and outgroup species used for phylogenetic analysis.

Species name	Voucher	Locality	COI	16S	H3	Reference
*Dendronotusalbus*	ZMMU:Op-566	USA: Washington	KX788135	KX788123	—	Korshunova et al. 2016
*Dendronotusarcticus*	ZMMU:Op-561	Russia: Laptev Sea	KX788140	KX788129	—	Korshunova et al. 2016
*Dendronotusclaguei*	LACM 3554	Mexico	—	—	MH756144	Valdés et al. 2018
*Dendronotusdalli*	ZMMU:Op-330	Russia: Kamchatka	KM396999	KM397081	KM397102	Ekimova et al. 2015
*Dendronotusfrondosus*	ZMMU:Op-380	Norway	KM396976	KM397056	KM397111	Ekimova et al. 2015
*Dendronotuselegans*	ZMMU:Op-269	Russia: White sea	KM396996	KM397078	KM397087	Ekimova et al. 2015
*Dendronotuseuropaeus*	ZMMU:Op-554	Norway	KY391823	KY391842	—	Korshunova et al. 2017
*Dendronotusiris*	CASIZ:174471	USA: Washington	KX058083	HM162631	HM162537	Ekimova et al. 2016a
*Dendronotusjamsteci*	JAMSTEC No. 1160047463	Japan	MN808558	MN811023	—	Martynov et al. 2020
*Dendronotuskalikal*	ZMMU:Op-283	Russia: Kamchatka	KC660024	KC611284	KC660044	Ekimova et al. 2015
*Dendronotuskamchaticus*	ZMMU:Op-245	Russia: Kamchatka	KC660032	KC611288	KC660048	Ekimova et al. 2015
*Dendronotuslacteus*	ZMMU:Op-584	Norway	KY391830	KY391849	—	Korshunova et al. 2017
*Dendronotusnordenskioeldi*	ZMMU:Op-665	Russia: Laptev sea	MT654636	MT655309	—	Korshunova et al. 2020
*Dendronotuspatricki*	SIO-BIC M12133	USA: California	HQ225828	HQ225829	—	Stout et al. 2011
*Dendronotusprimorjensis*	W196	—	KT031812	KT031825	MN138263	Ekimova et al. 2016b
*Dendronotusrobilliardi*	IE251	Russia: Kamchatka	KX058077	KX058117	KX058105	Ekimova et al. 2016a
*Dendronotusrobustus*	ZMMU:Op-343	Russia: Barents Sea	KM397002	KM397084	KM397106	Ekimova et al. 2015
*Dendronotusrufus*	LACM:174861	USA: Alaska	KX058084	GU339191	HQ267091	Ekimova et al. 2016a
*Dendronotussubramosus*	ZMMU:Op-699	USA: Washington	MN808564	MN811029	—	Martynov et al. 2020
*Dendronotusvelifer*	ZMMU:Op-348	Russia: Kara Sea	MF685027	KY996407	—	Lundin et al. 2017
*Dendronotusvenustus*	ZMMU:Op-660	USA: Washington	MK302460	MK302455	—	Korshunova et al. 2019
*Dendronotusyrjargul*	WS9116	Russia: Kara Sea	MN138317	MN138082	MN138232	Ekimova et al. 2019
*Dendronotuszakuro*	KSNHM: OP0485	Japan	MN808562	MN811027	MN138228	Martynov et al. 2020
*Pseudobornellaorientalis*	ZMMU: Op-664	Russia: Sea of Japan	MT654637	MT655310	—	Korshunova et al. 2020
*Pseudobornellaorientalis*	CASIZ:174989	China: Daisong Bay	—	HM162628	HM162534	Pola and Gosliner 2010
*Pseudobornellaorientalis*	**Op-24021903**	**China: Hailing Island**	**PV454581**	**PV457542**	**PV474719**	**This study**
*Pseudobornellaorientalis*	**Op-24021904**	**China: Hailing Island**	**PV454582**	**PV457543**	**PV474720**	**This study**
***Pseudobornellaqingdaoensis* sp. nov.**	isolate 01	China: Qingdao	OQ573562	—	—	Wei 2023
***Pseudobornellaqingdaoensis* sp. nov.**	**MBM288160**	**China: Qingdao**	**PV454583**	**PV457544**	**PV474721**	**This study**
***Pseudobornellaqingdaoensis* sp. nov.**	**MBM288155**	**China: Qingdao**	**PV454584**	**PV457545**	**PV474722**	**This study**
***Pseudobornellaqingdaoensis* sp. nov.**	**MBM288156**	**China: Qingdao**	**PV454585**	**PV457546**	**PV474723**	**This study**
***Pseudobornellaqingdaoensis* sp. nov.**	**MBM288157**	**China: Qingdao**	**PV454586**	**PV457547**	**PV474724**	**This study**
***Pseudobornellaqingdaoensis* sp. nov.**	**MBM288158**	**China: Qingdao**	**PV454587**	**PV457548**	**PV474725**	**This study**
***Pseudobornellaqingdaoensis* sp. nov.**	**MBM288159**	**China: Qingdao**	**PV454588**	**PV457549**	**PV474726**	**This study**
*Cabangusregius*	CASIZ:179492	Philippines	HM162708	HM162629	HM162535	Pola and Gosliner 2010
*Cabangusregius*	CASIZ:179493	Philippines	JN869451	JN869407	JN869430	Pola et al. 2012
*Marioniaarborescens*	CASIZ:177578	Philippines	HM162722	HM162646	HM162554	Pola and Gosliner 2010
*Marioniablainvillea*	CASIZ:176812	Portugal	HM162721	HM162645	HM162553	Pola and Gosliner 2010

Sequences were aligned with MAFFT (Katoh and Standley 2013) using ‘G-INS-i (accurate)’ strategy and normal alignment mode. Separate analyses were conducted for COI (634 bp), 16S (401 bp), H3 (302 bp), and concatenated data (1328 bp). Ambiguously aligned fragments of 16S alignment were removed using Gblocks (Talavera and Castresana 2007). Maximum-likelihood phylogenies (ML) were inferred using IQ-TREE (Nguyen et al. 2015) under Edge-unlinked partition model for 10,000 ultrafast bootstraps (Minh et al. 2013), as well as the Shimodaira–Hasegawa-like approximate likelihood ratio test (Guindon et al. 2010). Bayesian-inference (BI) phylogenies were inferred using MrBayes v. 3.2.6 (Ronquist et al. 2012) under partition model (2 parallel runs, 5 million generations), in which the initial 25% of sampled data were discarded as burn-in. The best-fit models of evolution (GTR+F+I+G4 for COI and 16S, GTR+F+I for H3) were determined by ModelFinder (Kalyaanamoorthy et al. 2017) using AIC criterion. Results were visualized using FigTree v. 1.4.3. The *p*-distances within and among each species grouping were estimated with MEGA 6 (Tamura et al. 2013) based on the Kimura 2-parameter (K2P) model (Kimura 1980).

### ﻿Species delimitation

The Automatic Barcode gap Discovery (ABGD) (Puillandre et al. 2012) and the Assemble Species by Automatic Partitioning (ASAP) (Puillandre et al. 2021) methods were used to assess the number of *Pseudobornella* species. The alignment from the fast-evolving COI gene was uploaded to the online servers of ABGD (https://bioinfo.mnhn.fr/abi/public/abgd/abgdweb.html) and ASAP (https://bioinfo.mnhn.fr/abi/public/asap), respectively. The analyses were performed with the model of Jukes-Cantor (JC69) with default settings.

## ﻿Systematics

### ﻿Order Nudibranchia Cuvier, 1817

**Superfamily Dendronotoidea Allman, 1845**

**Family Dendronotidae Allman, 1845**

#### ﻿Genus Pseudobornella

Taxon classificationAnimaliaNudibranchiaDendronotidae

Baba, 1932

89D7A121-500F-51C8-847A-0CC4EA4313B1

##### Type species.

*Pseudobornellaorientalis* Baba, 1932, by original designation.

##### Type locality.

Kanagawa Prefecture, Japan.

#### ﻿ Pseudobornella qingdaoensis sp. nov.

Taxon classificationAnimaliaNudibranchiaDendronotidae

B7FCCAAF-DFD7-5A81-ADA0-B2EF6C9CAA80

https://zoobank.org/3213D127-1786-4FA9-9BE7-9FEE128E1538

Figs 1 , 2 , 3

##### Type material.

**Figure 1.**
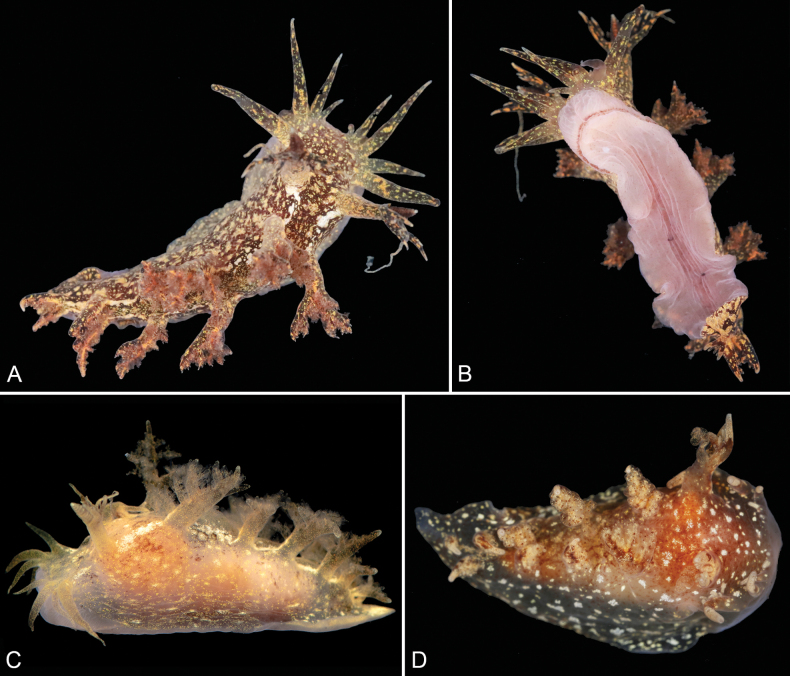
*Pseudobornellaqingdaoensis* sp. nov. **A, B** holotype, MBM288155, length 40 mm **C** paratype 5, MBM288160, preserved length 1.0 cm **D** paratype 6, MBM288161, preserved length 20 mm.

***Holotype*** (Fig. 1A, B): China • 1 specimen, length alive 40 mm; dissected; Shandong Province, Qingdao, Golden Beach Park, intertidal zone; 35°57'03"N, 120°14'26"W; 18 Jan. 2025; Shu-Qian Zhang leg.; MBM288155. ***Paratypes 1–4***: China • 4 specimens, lengths 35–40 mm; complete; complete; Shandong Province, Qingdao, Golden Beach Park, intertidal zone; 35°57'03"N, 120°14'26"W; 20 Jan. 2025; Shu-Qian Zhang leg.; MBM288156–MBM288159. ***Paratype 5*** (Fig. 1C): China • 1 specimen, preserved length 10 mm; dissected; Shandong Province, Qingdao, Taiping Bay, intertidal zone; 11 Apr. 2024; Ju-Hao Wang leg.; MBM288160. ***Paratype 6*** (Fig. 1D): China • 1 specimen, preserved lengths 20 mm; dissected; Shandong Province, Rushan, intertidal zone; 19 Apr. 2019; Ju-Hao Wang leg.; MBM288161. ***Paratypes 7–10***: China • 4 specimens, preserved lengths 20 mm; complete; same collection data as paratypes 6; MBM288162–MBM288165.

##### Diagnosis.

A large *Pseudobornella* reaching 40 mm in body length. Background color generally translucent yellow to dark brown, with numerous scattered orange to brown spots and white blotches. Radula formula 11 × 3.0.3.

##### Description.

External morphology (Fig. 1). Living animals up to 40 mm in length. Foot wide and tapering relatively abruptly towards the tail. Background color generally translucent yellow to dark brown, with numerous scattered orange to brown spots and white blotches. In some individuals, the white blotches forming two longitudinal streaks running along both sides of the dorsum. Anterior margin of head rounded. Each side of the mouth equipped with four or five smooth, tapering oral tentacles of varying size. Rhinophore sheath very elongate. Upper edge of each rhinophore sheath bearing four lateral digitiform branched papillae and one posterior papilla that is remarkably longer. Rhinophores with ~12 lamellae. Dorsal margin with four pairs of dorsolateral papillae, decreasing in size towards the posterior end of the foot. Each papilla with numerous unbranched gills attached to its inner side. Anus small, located on the right side of the dorsum between the first and second pair of dorsolateral appendages. Genital opening located on the right side, midway between the rhinophore sheath and the first dorsolateral appendage. Foot pale pink, scattered with numerous very small, indistinct darker spots, separated from the head by a long, transversal groove.

**Figure 2.**
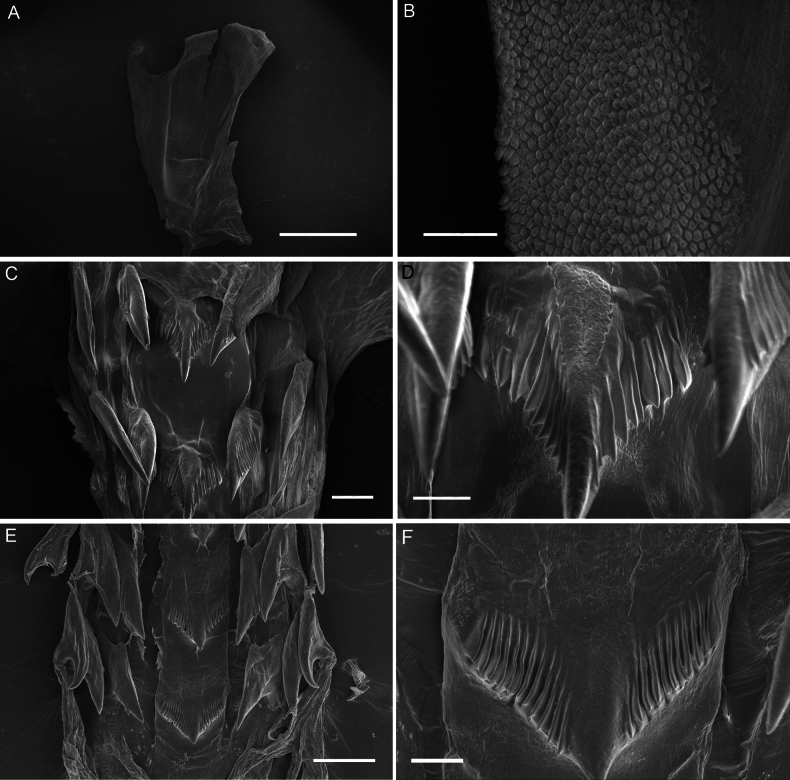
Jaws, radulae of *Pseudobornellaqingdaoensis* sp. nov. **A** jaw, MBM288155 **B** jaw masticatory border, MBM288155 **C, D** radula, MBM288155 **E, F** radula, MBM288161. Scale bars: 1 mm (**A**); 50 μm (**B, C**); 20 μm (**D, F**); 100 μm (**E**).

Internal anatomy. Jaws (Fig. 2A, B) elongate, inner margin (masticatory margin) terminating distally in a small, pointed process. Masticatory margin equipped with rows of short, cone-shaped rodlets. Radular (Fig. 2C–F) formula 11 × 3.0.3. Rachidian teeth stout, with a strong and bluntly pointed cusp and ~9–12 denticles on both sides of the cusp. Innermost lateral teeth with a very strong elongate and sharp cusp and serrated sides. Outer lateral teeth simple and hamate, and outermost one smallest.

**Figure 3.**
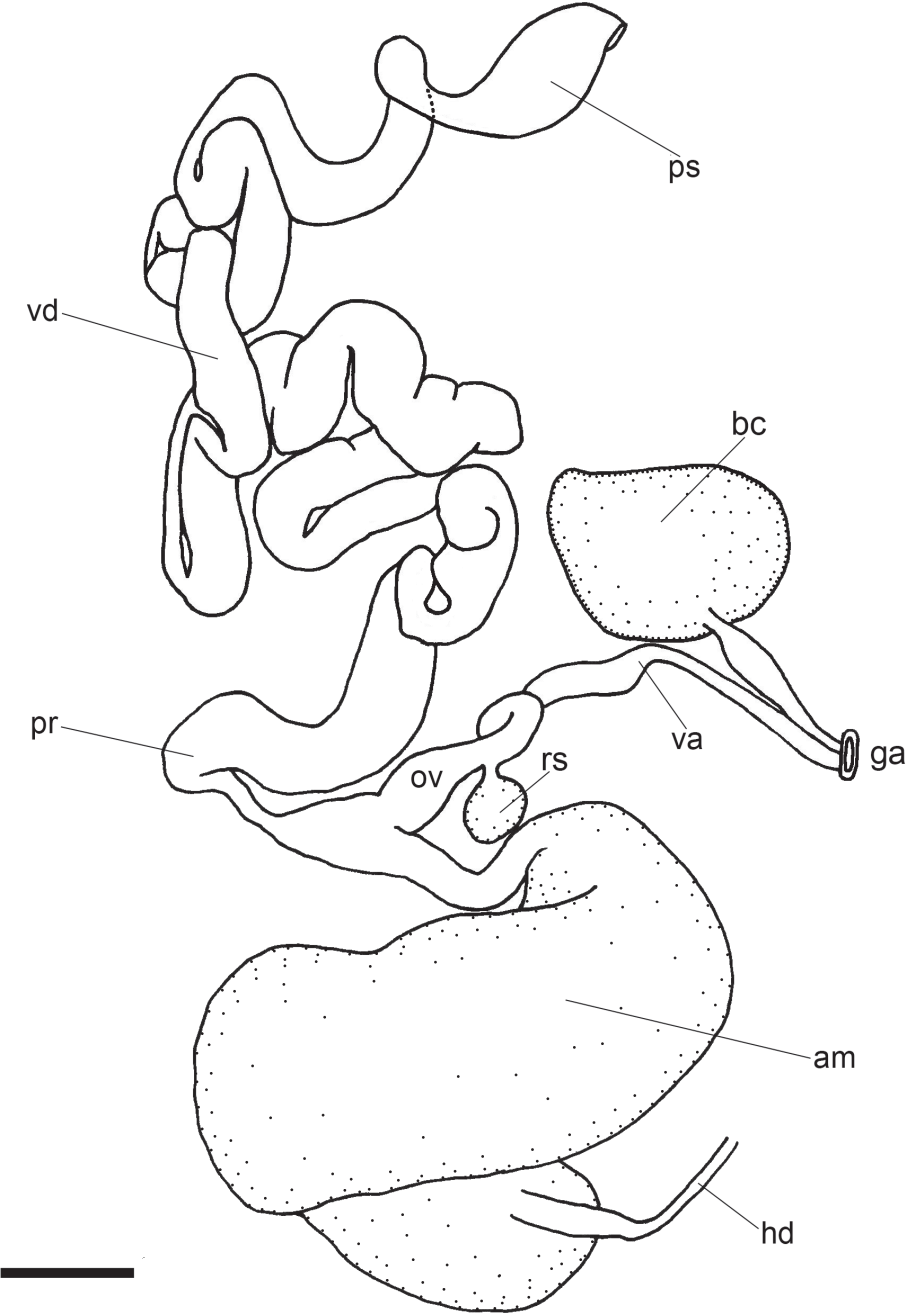
Reproductive system of *Pseudobornellaqingdaoensis* sp. nov., with ovotestis and female gland mass removed, MBM288155. Abbreviations: am–ampulla; bc–bursa copulatrix; ga–genital aperture; hd–hermaphrodite duct; ov–oviduct, pr–prostate, ps–penial sac; rs–receptaculum seminalis; va–vagina; vd–vas deferens. Scale bar: 1 mm.

Reproductive system (Fig. 3) located in the front right corner of the body. Ampulla very large, sausage-shaped, one end connecting with ovotestis via a thin hermaphroditic duct, the other end divided into two different, thin ducts: the short oviduct entering the female gland mass and the second duct branching into a wide, short prostate which extends into a long, folded vas deferens, narrowly opening to a wide and stout penial sac. Two ducts departing from the genital aperture: vagina relatively long and simply folded, connecting with a small, rounded receptaculum seminis; second duct wider and shorter, connecting with a very large pyriform bursa copulatrix.

##### Etymology.

The new species is named after its type locality, Qingdao.

##### Distribution and habitat.

Known from Yellow Sea, China, and possibly also distributed in Japan (Kinoshita 2002; Nobuhiko 2017). It was found in the rocky intertidal zone, feeding on a hydroid (Kinoshita 2002).

### ﻿Molecular support

**Figure 4.**
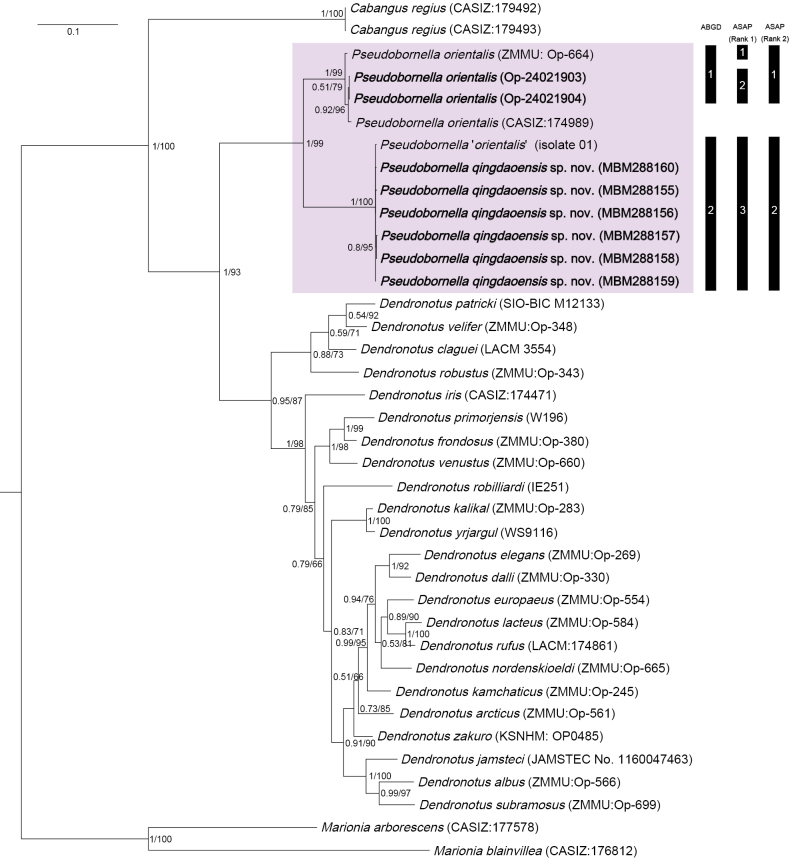
Phylogenetic tree inferred by Bayesian analysis (BI) and maximum likelihood (ML) based on concatenated dataset of COI, 16S and H3 genes. Numbers adjacent to nodes refer to BI posterior probability (PP > 0.5) and ML bootstrap scores. Numbers in the vertical black bars indicate the results of ABGD and ASAP species delimitations.

The phylogenetic trees inferred using BI and ML criteria were generally congruent (Fig. 4). The genus *Pseudobornella* was recovered as monophyletic with high support (PP = 1, BS = 99). Within the genus, *P.qingdaoensis* sp. nov. formed an independent clade (PP = 1, BS = 100) from *P.orientalis*. This result supports the systematic placement of the new species in the genus *Pseudobornella* and its separation from *P.orientalis*.

The ABGD analysis resulted in the delimitation of two species. The prior maximal distance (*p*-distance) ranged between 0.0017 and 0.1. However, the ASAP analysis recovered three partitions with the lowest score (generally considered to be the most supported partition scheme) (Fig. 4; Suppl. material 1). The partition with the second highest score was chosen here as it avoided the splitting of two populations of *P.orientalis* with a pairwise genetic distance of only 0.6%. In both cases, *P.qingdaoensis* sp. nov. was separated from *P.orientalis*.

Based on the available molecular data, the analysis of a 634-bp fragment of the COI gene yielded a pairwise distance of 11.7–12.5% between *P.qingdaoensis* sp. nov. and *P.orientalis*, a divergence much higher than the known intraspecific variation (0–0.6%) (Suppl. material 2), further supporting the separation of *P.qingdaoensis* sp. nov. from *P.orientalis*.

## ﻿Discussion

In this study, we incorporated the recently obtained molecular data for *Pseudobornellaorientalis* and the new species described here into the phylogenetic analyses. The results show that the family Dendronotidae was divided into three distinct clades with high support, corresponding to the three genera currently recognized: *Dendronotus* Alder & Hancock, 1845; *Pseudobornella* Baba, 1932; and *Cabangus* Korshunova, Bakken, Grøtan, K.B. Johnson, Lundin & Martynov, 2020. This topology is consistent with that of a previous study by Korshunova et al. (2020). Our results once again confirmed the validity of *Pseudobornella*. During the molecular analysis, we found a COI sequence of *P.orientalis* (accession number: OQ573562) derived from a specimen collected in Qingdao. However, this is a misidentification, as the external morphology of this specimen is clearly different from *P.orientalis* but consistent with *P.qingdaoensis* sp. nov. (photograph, Wei pers. comm., 7 Jun. 2024). The COI sequence of this specimen is almost identical to that of the new species, with only 0.2–0.5% divergence, further proving that they belong to the same species.

Despite the close resemblance between *P.orientalis* and *P.qingdaoensis* sp. nov., a comparison of their external morphology and internal anatomy allows for a clear differentiation between the two species. In terms of external coloration, the new species has prominent white spots scattered over the body surface and lacks the yellow diagonal stripes on the dorsal body surface that are characteristic of *P.orientalis*. In its internal anatomy, *P.qingdaoensis* sp. nov. differs from *P.orientalis* in having a distinct radula with three instead of two lateral teeth. Additionally, the vas deferens of *P.qingdaoensis* sp. nov. is significantly longer than that of *P.orientalis*. In Japan, some recorded individuals identified as *P.orientalis* are very similar to *P.qingdaoensis* sp. nov. in color pattern and number of oral tentacles (Kinoshita 2002; Nobuhiko 2017) and thus may be conspecific with the new species. If this is the case, the distribution of *P.qingdaoensis* sp. nov. would extend to Japan.

*Pseudobornellaorientalis* was first described in 1932 from Japan. Since then, no additional species have been described within the genus. A review of the literature and online photographic records suggests that the biodiversity of this group may be largely underestimated. As previously noted by Pola et al. (2009), some specimens identified as *P.orientalis* on the Sea Slug Forum (Rudman 2002) exhibit some differences, such as longer lateral papillae on the rhinophore sheath, indicating that these specimens may represent different species. Some naturalists have documented *P.orientalis* in the Northeastern Pacific (e.g. Johnson 2016; Pomeroy 2016; Young 2016; Agarwal 2017), but those specimens differ in the number of oral tentacles (five pairs) compared to those from the Western Pacific specimens (three pairs). The specimens are also similar to the new species by having five oral tentacles, but they differ in having a reticulated pattern of yellow stripes on the body surface and potentially represent another distinct species. Huang and Huang (2021) reported an undescribed species of *Pseudobornella* from Kinmen, China, but this species is easily distinguishable by its translucent whitish body with scattered purple-brown spots and oral tentacles with alternating purple-brown and white bands. Further integrative taxonomic work is needed to accurately determine the biodiversity of this group.

## Supplementary Material

XML Treatment for Pseudobornella

XML Treatment for Pseudobornella qingdaoensis

## ﻿Additional information

### Author ORCIDs

Shuqian Zhang https://orcid.org/0000-0002-1521-5317

### Data availability

All of the data that support the findings of this study are available in the main text or Supplementary Information.

## Supplementary materials

Supplementary material 1 ABGD analyses and ASAP analyses Data type doc https://binary.pensoft.net/file/1355858This dataset is made available under the Open Database License (http://opendatacommons.org/licenses/odbl/1.0/). The Open Database License (ODbL) is a license agreement intended to allow users to freely share, modify, and use this Dataset while maintaining this same freedom for others, provided that the original source and author(s) are credited.Shuqian Zhang, Juhao Wang

Supplementary material 2 Estimates of evolutionary divergence between sequences Data type xls https://binary.pensoft.net/file/1355859This dataset is made available under the Open Database License (http://opendatacommons.org/licenses/odbl/1.0/). The Open Database License (ODbL) is a license agreement intended to allow users to freely share, modify, and use this Dataset while maintaining this same freedom for others, provided that the original source and author(s) are credited.Shuqian Zhang, Juhao Wang
