# Supplementary figures and images for: ﻿Description of a new species of Pseudobornella Baba, 1932 (Gastropoda, Nudibranchia, Dendronotidae) from the Yellow Sea
Source: Zookeys. 2025 Jun 18;1241:301–14. doi: 10.3897/zookeys.1241.155540 (PMC12238979; doi:10.3897/zookeys.1241.155540)

**ABGD analyses:**


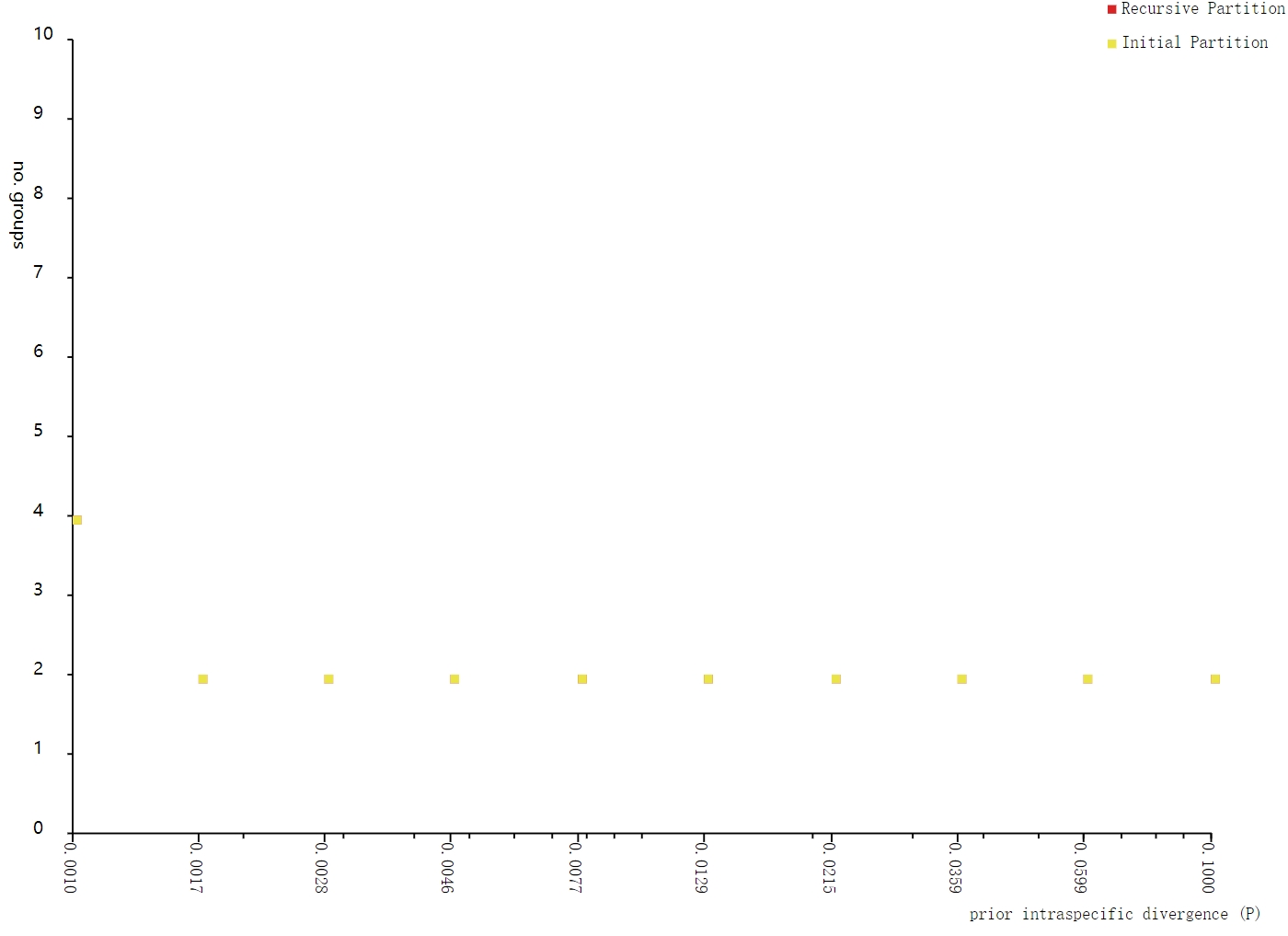

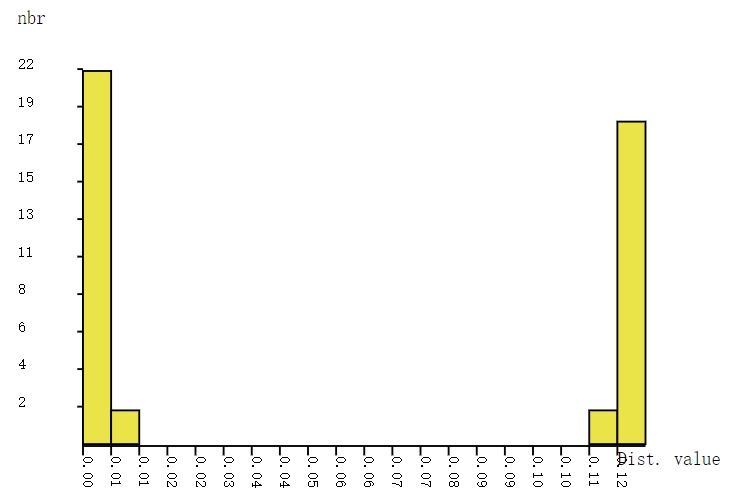


**ASAP analyses:**

**
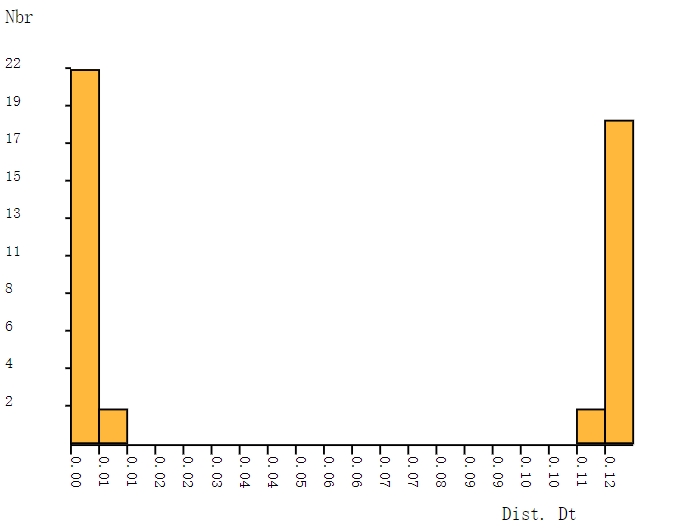
**

**
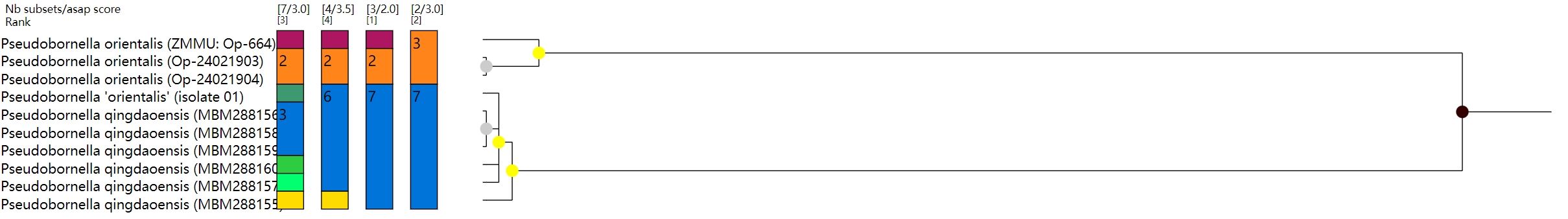
**

Supplement: Supplementary material 1 — ABGD analyses and ASAP analyses [file zookeys-1241-301_article-155540__-s001.doc]
